# Responsible digital transformation in rural primary healthcare services: critical factors from a Norwegian case study

**DOI:** 10.1080/02813432.2026.2673135

**Published:** 2026-05-23

**Authors:** Raj Kumar Thapa, Trond Bliksvær, Trude Fløystad Eines, Merete Kvamme Fabritius, Cecilie Katrine Utheim Grønvik, Solrun Holm, Nadra Jesmine Nilsen, Finn Robert Verlo, Trude Hartviksen

**Affiliations:** a Nordland Research Institute, Bodø, Norway; b Vestvågøy kommune, Leknes, Norway; c Molde University College, Molde, Norway; d Centre for Health Innovation, Kristiansund, Norway

**Keywords:** Home-based care, responsible digital transformation, rural municipality, qualitative case study, primary healthcare

## Abstract

**Objective:**

To explore the critical factors that shape the potential for co-creating and implementing a virtual department for home-based healthcare in a rural municipality.

**Methods:**

A qualitative case study was conducted in a rural municipality in Northern Norway. Data were collected through semi-structured interviews, focus groups, and co-creation workshops involving healthcare leaders, frontline staff, service users and relatives, and representative from the pensioners’ federation. Thematic analysis was applied to identify organisational, contextual, and cultural factors influencing digital transformation.

**Results:**

While the co-creation of virtual department for home-based healthcare showed potential for piloting, readiness for full implementation was limited. Six critical factors were identified: structural readiness, digital competence, technology in practice, cultural change, autonomy and care, and motivation and dedication. These factors highlight the importance of organisational culture, workforce capacity, and stakeholder engagement in shaping readiness for responsible digital transformation.

**Conclusion:**

Digital transformation holds potential for addressing healthcare challenges in rural municipalities, but success depends on more than technological efficacy. Effective coordination between bottom-up and top-down approaches is required to ensure responsible and sustainable transformation of primary healthcare services.

## 1. Introduction

Healthcare systems worldwide are under increasing pressure to deliver equitable and sustainable services in the face of rising demand for accessible, high-quality health and social care [1,2]. This pressure is exacerbated by an ageing population and the growing prevalence of chronic and complex conditions [3,4]. In Norway, the number of people aged 65 and older will surpass those aged 0–19 within the next decade, while the population aged 80 and above is projected to more than double by 2050 [5]. These demographic changes place significant strain on primary healthcare services, which municipalities are responsible for delivering [6].

Workforce shortages further intensify this strain, particularly in rural and remote municipalities where younger populations migrate to urban centres, leaving behind an ageing demographic [7]. This demographic imbalance is a well-documented challenge in rural healthcare globally, and in Norway it directly impacts the sustainability of municipal primary healthcare services [1]. Municipal strategies increasingly prioritize home-based care over institutional placements, as evidence shows that older adults are best supported in their own homes and that this approach is less resource-intensive [8–11]. Many older adults express a desire to remain at home, and this preference intersects with municipal strategies that prioritize home-based services. However, rural municipalities face persistent challenges in balancing rising demand for quality care with limited capacity [12,13]. Dispersed households and limited healthcare staff increase the burden on frontline workers, contributing to burnout and sick leave, which further strain municipal services [14]. To cope, municipalities often rely on temporary healthcare workers through staffing agencies, but this solution is economically unsustainable and raises ethical concerns [15]. Moreover, such alternatives are rarely viable in rural municipalities with limited resources and remote geography [16].

The COVID-19 pandemic further exposed systemic limitations and accelerated the urgency for innovative and sustainable alternatives [17–19]. Digital technologies offer promising avenues by enhancing the accessibility, efficiency, and quality of healthcare services [20,21]. However, the path to digital transformation is complex and requires careful consideration of societal needs, cultural values, stakeholder expectations, resource availability, and digital readiness [22–24]. Successful implementation also hinges on addressing concerns such as user privacy, data security, and the design of inclusive, user-cantered solutions [25].

To understand these complexities, we draw on Responsible Innovation (RI) as a conceptual background. Responsible digital transformation transcends the mere provision of digital technologies; it requires a critical and anticipatory approach to how technologies are designed and implemented, ensuring alignment with societal needs, values, and normative expectations. Accordingly, responsible digital transformation requires not only addressing user concerns but also fostering systemic change that contributes substantively to sustainable and socially desirable outcomes [27,28]. This perspective resonates strongly with RI, which emphasises that technological development must align with societal needs, values, and expectations, not only technical efficacy [29,30]. RI is commonly described through four interrelated principles: inclusion (engaging diverse stakeholders, including service users), anticipation (considering potential future impacts and risks), reflexivity (examining underlying assumptions and values), and responsiveness (adapting to emerging needs and concerns) [6,13,31,32]. These principles are particularly relevant in rural municipalities, where resource constraints, geographic dispersion, and infrastructural limitations demand transformation that is iterative, context-sensitive, and coordinated across micro, meso, and macro levels. In this study, RI does not guide the empirical analysis; rather, we engage it in the Discussion to situate the empirically derived domains within broader debates on responsible digital transformation.

A significant challenge in digital transformation lies in the top-down nature of many national digital strategies, which often fail to account for local needs, values, and capabilities [33]. When contextual factors such as demographic imbalances, workforce shortages, geographic dispersion, and limited digital infrastructure are overlooked, implementation efforts risk becoming inefficient or even counterproductive [33,34]. Moreover, numerous digital solutions are developed without meaningful involvement of end users, resulting in poor adoption and limited impact [27,30,35]. Empirical research consistently shows that health technology innovations frequently fall short because they neglect user motivation, adoption processes, and complex realities of implementation contexts [28,36].

Digital transformation in healthcare services therefore entails a fundamental reconfiguration of service delivery models, organisational practices, and stakeholder relationships [37]. It must be tailored to specific community contexts, as one-size-fits-all approaches are unlikely to succeed. While digital transformation holds great potential, realizing its benefits requires a nuanced and participatory approach that integrates local needs, values, and capacities.

Research question: **What contextual and organisational factors shape the potential for responsible digital transformation of healthcare services in a rural municipality?**

## 2. Methodology

### 2.1. Study design

This study employed a single case study design in which the case itself was the rural municipality in Northern Norway. The municipality constituted the bounded system under investigation, and the analysis examined how it worked to ensure inclusive healthcare provisions while pursuing digital transformation initiatives to strengthen service quality.

Single case studies are particularly valuable for investigating complex, context-dependent phenomena and for gaining an in-depth understanding of a unique organisational setting [38,39]. This approach was well suited to the municipality’s distinctive contextual challenges and its ongoing efforts to develop digitally supported home-based healthcare services.

### 2.2. Case description

The study took place in a rural municipality located on one of the islands in the Nordland County, Northern Norway. Norway operates within the Nordic welfare-state tradition, characterised by publicly funded services, universal entitlements, and a strong legislative framework. The municipality has a population of roughly 11,600 residents and covers an area of about 400 km^2^. Its administrative hub is located in a village with approximately 3,800 inhabitants, while the remaining population lives in smaller settlements scattered across the island, resulting in a density of around 29 residents per square kilometre. In 2022, 21% of the municipality’s around 5000 employed residents worked in the municipal sector [40].

Like many rural municipalities in Norway, this municipality faces challenges in delivering healthcare services, including shortages of healthcare professionals, increasing demand for equitable access to care, and strained municipal finances. To address these challenges, the municipality has initiated several strategic measures. One major initiative is Kunnskapskommunen Helse Omsorg Nord (‘The Knowledge Municipality Health Care North’), a collaborative network involving regional municipalities, Nord University, and UiT the Arctic University of Norway. The initiative aims to strengthen research, innovation, and knowledge development within municipal health and care services, ensuring that residents receive high‑quality, evidence‑based care.

The municipality is also involved in several research and innovation projects, including a ‘digital first choice’-project that started three years ago with the intention to explore the opportunities, limitations, and dilemmas associated with developing a virtual unit within home-based services in a rural and coastal municipality. Its aim has been to investigate what ‘digital first choice’ could practically mean for both patients and staff in home-based services in such a context, how it could be implemented through co-creation, and how a virtual unit could be integrated into the organisation as a whole.

In the healthcare service research, a virtual ward or virtual department refers to an organisational model that provides hospital-level care in patients’ homes through multidisciplinary teams supported by digital platforms [41,42]. The ‘digital first choice’-project of the case municipality represented an early-stage effort, that was originally operationalised through a digital platform intended to develop into a virtual unit integrated into municipal home-based services. As part of the learning process from the ‘digital first choice’-project, the municipal actors recognized that a reorientation was needed, that reflected a broader understanding of virtual wards as digitally enabled organisational forms - rather than physical departments moving from platform to unit and ultimately to department. It is this process of reorientation that has called for a broader theoretical framework, and which will be investigated in this paper through the lens of Responsible Innovation.

### 2.3. Data collection

The municipality was integrally involved in organising the activities of the action research project, in collaboration with external research partners. It coordinated workshops and managed participant invitations, supported by service designers from one of the external project partners of the ‘digital first choice’ project. Recruitment was facilitated by the municipal healthcare unit, which identified suitable participants across different subunits of the municipal healthcare services, including home-based care, rehabilitation and reablement services, administrative units, and departmental leadership.

Participants included frontline healthcare professionals (registered nurses and healthcare assistants), administrative staff, departmental leaders, service users, and relatives. This ensured representation from actors positioned at different organisational levels within the municipality.

### 2.4. Data collection process

The study employed a data triangulation strategy, combining various qualitative sources to ensure richness and credibility [43]. Data were collected through:

- Semi-structured individual interviews (n = 14): conducted both face-to-face (n = 11) and online (n = 2), each lasting 45–60 minutes.
  - Healthcare personnel (frontline professionals and departmental leaders)
  - Service users and relatives (Four individual interviews)
- Focus-group interviews (n = 5): involving healthcare personnel or department leaders, each lasting approximately one hour, with 2–8 participants per group.
- Co-creation workshops (n = 3): hosted by the municipality, with attendees including representatives from project partner institutions, the pensioners’ union, healthcare professionals, departmental leaders and members of the IT department.

An interview guide was developed prior to the interviews, tailored for different roles. Notably, interview guides for nurses and healthcare assistants included questions about their experience with service users, recognizing their direct interactions with patients and next of kin. Interviews were recorded and transcribed using AI-assisted transcription software and manually cross-checked for accuracy. The interview transcripts comprised a total of 520 A4 pages.

### 2.5. Data analysis

Data were analysed using thematic analysis, drawing on both the reflexive approach outlined by [44] and the phronetic iterative framework proposed by [45]. This combined approach enabled structured theme development while maintaining contextual sensitivity and practical relevance.

Data from individual interviews, focus groups and workshops were incorporated to enrich the analysis and enable data triangulation [43]. All data sources were analysed together rather than as separate subunits, reflecting the study’s aim to understand digital transformation as a system-level phenomenon spanning organisational boundaries. This integration supported dialogic validation of themes and contributed to findings that are both contextually rich and practically meaningful.

The data analysis process was led by two researchers from one of the external research partners (first and second author of the paper). One researcher from the municipality’s Research and Development (R&D) department took part in analysis through collective discussions and writing. Each researcher engaged in individual familiarization with the data, generating preliminary codes and analytic memos. These insights were then brought into a three-hour virtual analysis seminar, where the team collaboratively discussed emerging patterns, refined codes, and initiated theme abstraction. Findings were documented and circulated among the group, followed by a second virtual meeting to critically reflect on the coherence, relevance, and resonance of the developing themes [44]. These discussions supported the interpretive development of themes, rather than consensus-based validation, which aligns with the principles of reflexive thematic analysis.

During theme development, recurrent codes were not simply grouped by topic; rather they were examined for patterns of shared meaning organised around central organising concepts, consistent with reflexive thematic analysis. For example, the theme **Structural readiness** was developed from interpretive codes such as ‘framing digitalisation as structural change,’ ‘coordination structures enabling transformation’, and ‘workflow redesign for technologies’. When reviewed collectively, these codes reflected a deeper meaning pattern: participants consistently understood digital transformation as requiring system-level alignment and organisational reconfiguration. This shared meaning formed the central organising concept of the theme. These interpretive codes served as analytic tools rather than reporting units; in the findings, themes are illustrated through representative participant quotes that capture the broader meaning pattern rather than each individual code. Sub-themes (presented as key components in Table 1) captured specific practices, concerns, or enablers repeatedly emphasised by participants.

**Table 1.** Six domains and their corresponding key components.

Domain (overarching themes)	Key components (sub-themes)
System and Structure	Pilot framing as permanent shift Coordination meetings Workflow redesign for technologies
Digital competence	Upskilling staff *SeniorNet* and digital inclusion/ user support Shift towards *mastery-oriented* culture
Technology in practice	Predictive monitoring Usability feedback Embedded routines
Culture change	Interdisciplinary collaboration Trust-building Support for testing
Autonomy and care	Emotional reassurance Tailored solutions Respect for independence
Motivation and dedication	Professional pride Proactive efforts Professional commitment

This multi-source, multi-phase process enhanced analytical rigor and ensured that the final themes reflected diverse perspectives and organisational realities. Through this process, six analytic themes were identified: structural readiness, digital competence, technology in practice, culture change, autonomy and care, and motivation and dedication. These six analytic themes were subsequently conceptualised as broader domains, which together form the framework presented in the Discussion.

While the framework of Responsible Innovation (RI) informed the conceptual framing of this study, the six domains presented in the framework were derived inductively from empirical data through thematic analysis. RI principles—Inclusion, Anticipation, Reflexivity, and Responsiveness—were later used to interpret and situate the findings within broader debates on ethical and equitable digital transformation. This approach allowed for a practice-informed framework that aligns with RI values without being constrained by them.

### 2.6. Ethical considerations

Ethical approval for the study was obtained from SIKT^1^. All participants received written and oral information about the study and provided informed consent prior to participation. Participation was voluntary, and individuals could withdraw at any time without consequences. Data were anonymised during transcription and stored securely in compliance with GDPR regulations, and only the research team had access to the material.

Given the municipality’s active role in organising workshops and facilitating recruitment, we recognised the potential for gatekeeper influence and selection bias. Recruitment through municipal leaders may have favoured participants who were more engaged, more experienced, or perceived as supportive of ongoing initiatives. To mitigate this, we sought to include a broad range of stakeholders—frontline healthcare personnel, departmental leaders, service users, relatives, and workshops. Reflexivity was emphasised throughout the research process to critically examine how our collaboration with the municipality might share data generation an interpretation.

The study design respected participants’ autonomy and confidentiality, and findings were shared with the municipality to ensure transparency and reciprocity. Nonetheless, we acknowledge that the recruitment process may have influenced the perspective represented in the data, and this remains a methodological limitation.

## 3. Findings

This section presents six interrelated domains developed through reflexive thematic analysis of interviews, focus groups, and workshops involving healthcare leaders, frontline staff, service users, relatives, and other stakeholders in the municipal healthcare ecosystem. The analysis revealed how digital transformation was experienced across organisational levels and personal contexts, highlighting not only technical and procedural shifts but also cultural, emotional, and ethical dimensions of change.

Each domain captures a distinct, yet interconnected aspect of how digital tools are being integrated into municipal healthcare in rural Northern Norway. Together, they offer a nuanced understanding of what responsible digital transformation looks like in a geographically dispersed municipality illuminating both the opportunities and tensions that arise when technology intersects with healthcare.

### 3.1. Structural readiness

The first theme emerged from the results when both the participating healthcare leaders and personnel consistently emphasised the importance of structural readiness and system-level coordination as prerequisites for successful digital transformation. Leaders described the importance of not framing pilot projects like temporary experiments, but as long-term shifts in service delivery. As one healthcare department leader explained during a focus group:

I have deliberately avoided framing this as a project or a pilot initiative. Rather, I regard it as a lasting transformation that is here to stay. (Leader, focus group)

Healthcare workers highlighted the need for clear routines, shared digital platforms, and aligned procedures across departments to ensure consistency and quality. Interdisciplinary meetings, known locally as *‘whiteboard meetings’,* were described as essential arenas for bringing together diverse professional perspectives, fostering mutual understanding, and supporting collaborative decision-making. These meetings were described by the healthcare workers as a key structural tool for anchoring and driving change processes. One nurse reflected:

When the leader isn’t present at the board huddle, the dialogue flows more easily. The discussions are more fruitful. (Staff 1)

Beyond the more formal whiteboard meetings, staff described informal coordination practices that helped avoid duplication of tasks and fostered shared responsibility for follow-up and care continuity. For example, one rehabilitation worker explained how her team proactively initiated contact with home care services:

We drop by the home care unit during lunch and talk about the users we have in common… and they can refer new ones to us. (Staff, home based reablement)

These structural mechanisms were not only essential for service coordination, but they also laid the groundwork for integrating welfare technologies. Participants described how digital tools like Sleep monitors (*Somnify)* and Personal Emergency Response System required not just technical infrastructure, but also organisational restructuring. This included clarifying roles, redesigning workflows and adapting routines to accommodate new forms of monitoring and response. For example, staff noted that *Somnify’s* alerts had to be embedded into existing care pathways, and that digital alarms required clear protocols for escalation and follow-up. This was supported by one relative that also worked as a leader in the municipal healthcare:

At first, only I received the alerts from Somnify. Later, the system was connected to the night patrol. It had to be integrated into their routines. (Leader)

Both the participating health personnel, leaders and user representatives provided statements that clarified that for them, digital transformation was not simply about adopting new tools—it was about reconfiguring the system to support their meaningful use. Structural readiness, leadership flexibility, and cross-sector collaboration were all seen as essential to making welfare technology work in practice.

### 3.2. Digital competence

Digital competence emerged as a critical enabler of transformation across all stakeholder groups. Healthcare staff expressed both enthusiasm and concern about mastering new digital tools and platforms, while department heads emphasized the need for ongoing training and support as technology becomes increasingly embedded in everyday practice.

You can’t just start using welfare technology—you need involvement and structural change first. (Leader)

The analysis also underscored the importance of including service users’ needs and expectations in digital transformation efforts. Many older users relied heavily on family members or healthcare staff to bridge digital gaps, highlighting the significance of intergenerational support and intuitive design. One user illustrated this when she described how a family member helped her connect a hearing aid to her mobile device:

My family member connected a hearing aid to my mobile device, enabling my access to live-streamed church services from home. This technological solution has greatly enhanced my listening experience, making the content clearer and more accessible than in a physical set.

Initiatives like senior learning networks and local training sessions for elder people helped build digital literacy, but uptake remained limited. Moreover, rear and unfamiliarity with technologies were common barriers among older users. As one community leader expressed:

Out of 2,000 older residents in the municipality, maybe only 300 are digital. Many of us older adults are hesitant to embrace digital technology’. They’re afraid to press the wrong button… or that it might explode. (Leader pensioners’ union).

Participants further emphasized the professionalization of care, reflected in a shift in professional language—from pleie *og omsorg* (‘care and kindness’) to *mestring* (‘mastery’)—reflecting a broader cultural change towards goal-oriented, respectful, and empowering service delivery.

Despite these positive momentums, several challenges were identified. These included the absence of a municipal digitalization strategy, insufficient onboarding routines for staff, and persistent digital exclusion among older users. Motivation and tailored support were seen as essential for ensuring inclusive and equitable transformation.

### 3.3. Technology in practice

Stakeholders including healthcare professionals, service users, and relatives expressed that when technology functions effectively and serves its intended purpose, it becomes a natural part of day-to-day healthcare routines. Healthcare professionals noted that digital tools can enhance patient safety, reduce unnecessary home visits, and enable predictive monitoring. Both users familiar with technology and healthcare staff described how these tools supported autonomy while offering emotional reassurance. As one relative described his satisfaction with the assistance of technology in reframing conversations about sleep quality:

Mum said she slept poorly, but the data showed 90% sleep quality. Now we can talk about what’s really happening. (Relative).

Similarly, others expressed how monitoring technology enabled early detection of health changes and medication effects. These tools were not only reactive but increasingly predictive—allowing healthcare professionals and relatives to adjust routines, medication timing, and follow-up based on real-time or near-real-time data. For example, one participant described how Somnify data helped delay morning medication when the user had slept poorly:

Based on the available data, I am able to inform her that the records indicate she was awake twice during the night—at approximately [time] and [time]—and that she returned to bed and fell asleep quickly, with subsequent sleep characterized by high quality.

However, participants also reflected on usability challenges, such as device placement, sensor sensitivity, and inconsistent functionality. These issues highlighted the need for adaptive solutions and user-cantered design to ensure that technology remains accessible, reliable, and responsive to individual needs.

Connectivity was another concern, particularly in rural areas with variable internet coverage. While most homes had stable broadband, some required mobile routers or fallback SIM cards. These reflections underscore that successful and responsible digital transformation depends not only on the availability of technology, but on its thoughtful integration into everyday care with attention to physical environments, user habits, and infrastructure constraints.

### 3.4. Cultural change

Stakeholders emphasised that digital transformation must be accompanied by a gradual cultural shift involving healthcare professionals, service users, relatives, and technology providers. Leaders noted that prior involvement in improvement initiatives helped normalise change and foster readiness for innovation. Staff described a growing openness to technology and interdisciplinary collaboration. One staff member explained how her team initiated regular coordination with home care services:

We’ve started meeting monthly with home care…to coordinate efforts and avoid duplication.

Service users, particularly older adults, expressed willingness to test new tools when introduced with empathy, support, and clear relevance to their daily lives. A relative described how his mother’s initial scepticism gave way to trust:

She called it a ‘dippe dutt’ at first, but now she sees it as a smart safety net. (Relative)

During the project period, a sort of maturing process was described, a shift that involved different actors and their evolving understanding of the process they were part of. Among the leaders, attention was given to the concept of leadership and how leadership may promote positive change. One leader made this reflection:

I care about change—not for the sake of change itself but change that actually leads to improvement. To achieve that, we need a shared understanding of the problem; only then can we start discussing solutions. It’s important to focus on participation and to establish a culture of feedback where employees feel free to express whether they agree or disagree. (Leader)

This shift in both leadership attitude and organisational culture emphasizing participation, feedback, and inclusion was reinforced by practical success and emotional reassurance. In particular participants highlighted the importance of inclusion in decision-making processes, which contributed to a sense of ownership and the emergence of a ‘we-feeling’ across departments. These reflections illustrate a cultural change from resistance to engagement, as staff began to value collaboration and feedback.

We’ve involved staff across departments in workshops… they say it’s good to talk to each other in new ways. (Leader)

At the same time, participants noted some technologies remained underutilised due to lack of strategic direction and staff-training. While certain tools were widely accepted, newer technologies required more structured onboarding and cultural adaptation. Participants including healthcare professionals and relatives of care recipients emphasized the importance of practical aids to make technology approachable. One relative illustrated this by describing how visual instructions and laminated sheets helped to demystify the technology::

We hang a laminated sheet on the bedroom door with a QR code that links to a simple explanation of what Nattugle is. (Relative of care recipient)

This example reflects a broader move from hesitation to engagement, where practical solutions supported confidence and cultural adaptation across different user groups.

### 3.5. Autonomy and care

Stakeholders reflected on their values, concerns, and expectations regarding the digital transformation of healthcare services. Service users emphasised the importance of independence and dignity, valuing technology not solely for its functionality but for the emotional security it provided. One user aged 92, described her continued sense of agency despite physical limitations:

I’m strong from the waist up… I’ve worked all my life, raised seven children, and I still manage most things myself. (User)

Digital tools enabled users to remain safely at home while reducing the need for intrusive nighttime visits. These technologies were especially appreciated when introduced with family support and personalised setup as one of the relatives expressed:

She knows Somnify is watching over her…but she almost forgets it’s there.

Family members played a critical role in interpreting data and advocating for tailored support. However, both staff and users expressed concerns that technology might replace the human touch central to caregiving. While digital tools were welcomed as supportive aids, participants cautioned against scenarios where they might displace compassionate, hands-on care:

As long as technology is used as assistance, it’s positive—but if it replaces warm hands, it’s alarming. (Staff reflection)

These insights underscore the need for a balanced approach to digital transformation, one that enhances autonomy without compromising the relational and emotional dimensions of care.

### 3.6. Motivation and dedication

Despite operational pressures and increasing demands, participants expressed a strong desire to contribute meaningfully to the transformation of healthcare services. Leaders and staff described a sense of pride in their work, emotional resilience, and a commitment to continuous improvement:

You just have to hold on. It takes time, but it will be worth it. (Leader)

However, staff also voiced concerns about workload accumulation and the lack of task reprioritization, which risked undermining morale and sustainability:

We never hear: ‘Now that you’re doing this, you can stop doing that.’ It just keeps piling up. (Staff)

Still many described proactive efforts to improve care, such as initiating meetings with general practitioners or adapting routines to better support interdisciplinary collaboration.

These reflections suggest that while intrinsic motivation remains strong, sustaining engagement and preventing burnout requires structural support and recognition. Responsible digital transformation must therefore encompass not only technological and procedural change, but also sustained attention to the emotional and professional wellbeing of those who deliver care.

Table 1 provides a concise overview of the six themes, summarising their key components and the recurrent practices described by participants.

## 4. Discussion

The digital transformation of healthcare is widely recognised as both necessary and fraught with complexity [6]. Current debates emphasise the tensions between technological innovations and systemic readiness, the risk of exacerbating inequalities, and the need for human-cantered, ethically grounded approaches [46]. While global and national strategies promote interoperability, equity, and sustainability (e.g. Ref. [47], Norwegian Directorate of eHealth, 2024), empirical studies continue to highlight persistent gaps in workforce capacity, infrastructure, organisational culture, and local adaptation [27,30].

Our findings contribute to this debate by offering a context-sensitive, empirically grounded framework that foregrounds the lived experiences of healthcare professionals, and stakeholders in a rural municipality in Northern Norway. Unlike dominant models that prioritise infrastructure, data governance, or digital capabilities, our framework emphasises relational dynamics, professional motivation, and cultural adaptation as core components of responsible digital transformation.

Specifically, our framework addresses three unexplored tensions in the literature:

- **Responsibility beyond compliance**: Digital transformation is often framed as a technical or regulatory upgrade [48,49]. However, our findings reveal that emotional reassurance, professional pride, and ethical alignment are central to how care professionals understand and enact responsibility, particularly within the domains of *autonomy and care, and motivation and dedication.* This resonates with recent studies highlighting the importance of emotional and psychological safety in digital healthcare [50,51], trust and ethical alignment in digital systems [52,53], and the expectations of professionals for leadership that sustains professional identity and pride [54].
- **Transformation as iterative and embedded:** Within the domain of structural readiness, pilot initiatives frequently evolve into enduring organisational shifts through mechanisms such as coordination, workflow redesign, and adaptive governance. Rather than representing a one-off intervention, digital transformation unfolds as an iterative and reflexive process that becomes deeply embedded in everyday practice. This interpretation is consistent with studies emphasising the progressive institutionalisation of pilot projects into routine structures [55], the significance of adaptive governance in sustaining digital health initiatives [49], and the need for continuous workflow reconfiguration to embed innovation in practice [48].
- **Peripheral context as sites of innovation**: Rural municipalities are often portrayed as lagging in digital adoption, framed through deficit-based narratives of limited resources and infrastructural constraints [56]. Yet our data shows proactive efforts, interdisciplinary collaboration, and tailored solutions, particularly within *culture change, technology in practice,* and *digital competence.* Rather than sites of delay, peripheral contexts demonstrate innovative potential and adaptive capacity. This interpretation resonates with studies emphasising the creative agency of rural actors in digital innovation [56, Eines et al, 2023), the role of interdisciplinary collaboration in overcoming structural barriers [57], and the importance of tailored competence-building for professional in peripheral regions [58].

Through the lens of Responsible innovation (RI), a holistic and dynamic approach to digital transformation can be conceptualised, enacted, and sustained. The RI principles inclusion, anticipation, reflexivity, and responsiveness do not function as abstract ideals but as practical orientations shaping how technologies are designed, implemented, and adapted [6,59,60]. In our study, these principles surfaced not as isolated actions but as embedded values across the six emerging domains, consistent with applied RI in digital health and ICT contexts [61,62]. For example, inclusion was evident in user-led training and interdisciplinary collaboration; anticipation emerged through predictive monitoring and usability feedback; reflexivity was reflected in workflow redesign and cultural shifts; and responsiveness was demonstrated through tailored solutions and the ability to pivot when technologies failed to meet expectations. These expressions of RI were not linear or uniform; they varied across contexts and evolved through interaction, reflection, and adaptation, echoing findings from complexity-oriented health services research [55].

The six domains identified through thematic analysis: structural readiness, digital competence, technology in practice, culture change, autonomy and care, and motivation and dedication represent practice-based expressions of RI in rural healthcare. Each domain comprises key components that reflect the needs, concerns, and aspirations of stakeholders, and together they form the foundation of a dynamic, iterative framework for transformation [48]. Our findings also reinforce the importance of multi-level coordination across micro) individual), meso (institutional), and macro (policy) levels and a balanced mechanism of bottom-up and top-down approaches. Bottom-up strategies foster societal readiness, encompassing both technological capacity and social efficacy, while inclusive development processes allow for the reflection of stakeholder needs and enable anticipation of opportunities, consequences, and barriers [63,64].

Moreover, responsible transformation demands a shift in organisational culture, including critical reflection on the purpose, process, and outcomes of digital initiatives [6]. It requires supportive infrastructure, digital literacy among both care providers and recipients, and collaborative engagement across the ecosystem. Reflexivity in communication and transparency about anticipated consequences and mitigation strategies are vital for building user confidence and strengthening trust [49, P. 53]. Following changes in organisational conditions, digital tools become embedded as an inherent component of practice, rather than functioning as an added layer of activity, underscoring the importance of cultural adaptation and readiness in sustaining transformation [48].

Stakeholders must also develop responsive capabilities, the ability to adopt change when it aligns with expectations and values, and to pivot toward alternatives when it does not [25]. This dynamic adaptability is central to sustaining meaningful transformation and achieving long-term impact [65,66].

While analytically distinct, the six domains operate as an interconnected system rather than isolated findings. Structural readiness provides the organisational scaffolding for digital tools; digital competence shapes the capacity of staff and users to engage with them; technology in practice captures how tools are negotiated and adapted in everyday work; culture change enables the relational and behavioural shifts required for sustained adoption; autonomy and care articulate the ethical and emotional dimensions shaping acceptance; and motivation and dedication sustain engagement over time. These domains reinforce one another dynamically, and it is this relational interplay rather than the presence of any single domain that constitutes the robustness of the framework. This integrated configuration distinguishes the model from existing approaches that focus primarily on technological or infrastructural readiness.

To consolidate these insights, we present an iterative and interactive framework that captures the dynamic, relational, and evolving nature of responsible digital transformation in municipal healthcare services. This framework reflects how the six domains interact with the principles of Responsible Innovation and directional coordination approaches, offering a practice-informed roadmap for coordinated, multi-level change [6,55].

**Figure 1.**
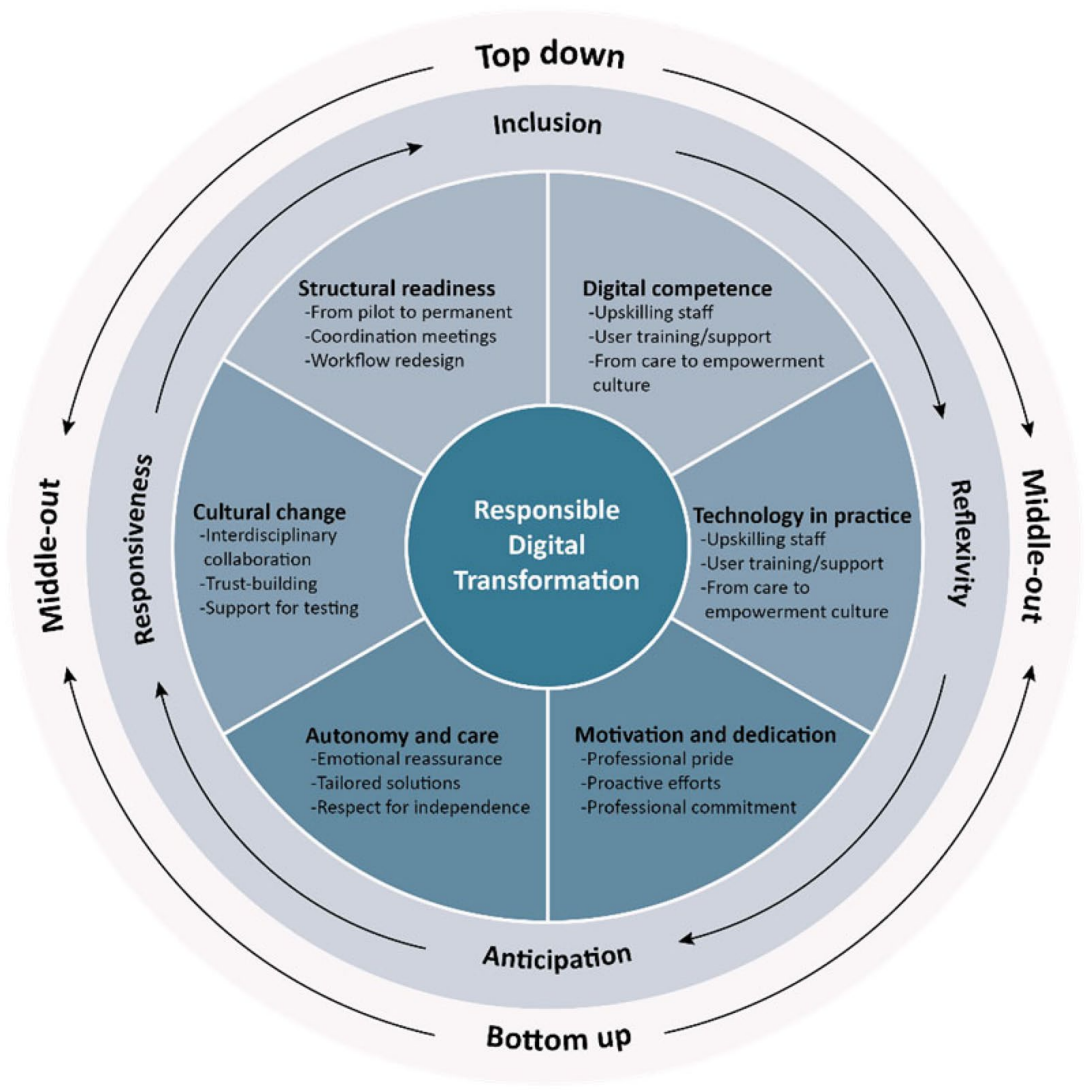
Framework for responsible digital transformation in municipal healthcare services.

This is illustrated in Figure 1, which presents the full framework.

The circular framework integrates six empirically derived domains: structural readiness, digital competence, technology in practice, culture change, autonomy and care, and motivation and dedication with the principles of Responsible Innovation (Inclusion, anticipation, reflexivity responsiveness) and directional coordination approaches (top-down, bottom-up, middle-out). In addition to top-down and bottom-up dynamics, the framework incorporates a middle-out perspective. Middle-out refers to the coordinating influence of mid-level actors such as department heads, project coordinators, and service leaders who translate strategic goals into operational practice while also channelling frontline insights upward. These actors play a bridging role that enables iterative adaptation and alignment across organisational levels. The structure illustrates the dynamic, relational, and iterative nature of transformation across multiple levels of the healthcare ecosystem.

## 5. Conclusion

This study presents an iterative, interactive, and dynamic framework for responsible digital transformation in rural healthcare. Grounded in the lived experiences of professionals and stakeholders, the framework comprises six interrelated domains: Structural readiness, Digital competence, Technology in practice, Culture change, Autonomy and care, and Motivation and dedication. Together, these domains reflect the multifaceted realities of transformation, encompassing organisational, technological, cultural, and emotional dimensions.

Our contribution lies in three key areas: First, the framework is empirically grounded, derived inductively from qualitative data, and shaped by the voices of those directly involved in service delivery. Second, it is contextually sensitive, challenging deficit-based narratives about rural municipalities and highlighting their capacity for innovation, adaptation, and leadership. Third, it is process-oriented, capturing the dynamic and evolving nature of transformation through continuous coordination, stakeholder engagement, and feedback loops across micro (individual), meso institutional), and macro (policy) levels.

Several limitations must be acknowledged. The study is based on a single rural municipality in Northern Norway, and while richly contextualised, its findings may not directly apply to other settings without adaptation. Additionally, the framework reflects stakeholder perspectives at a particular moment in time; as technologies, policies, and societal expectations evolve, so too may the relevance and expression of these domains.

Future research should explore the applicability of this framework across diverse geographic and institutional settings, including urban and cross-sectoral contexts. Longitudinal studies could examine how these domains evolve over time and in response to policy changes, technological advances, or cultural shifts. Further work is also needed to operationalise RI principles within digital transformation initiatives, particularly in ways that support anticipation, reflexivity, responsiveness, and inclusion across all levels of the system.

The study offers transferable insights that may inform responsible transformation in similar contexts. While the framework aligns conceptually with the principles of Responsible innovation (RI), RI did not guide data collection or coding; its role in this study is interpretive rather than analytical. Taken together, these insights underscore the importance of relational, contextual, and iterative approaches to digital transformation in municipal healthcare services Future research may further test, refine, or adapt this framework across diverse settings, supporting the development of more responsive, equitable, and sustainable digital healthcare practices. Taken together, these insights underscore the importance of relational, contextual, and iterative approaches to digital transformation in healthcare. Responsible digital transformation is not a one‑time intervention but a dynamic, evolving process. This framework provides a foundation for navigating that complexity and advancing meaningful, equitable innovation in healthcare.

Ultimately, responsible digital transformation is not a one-time intervention but a dynamic, iterative, and interactive process of systemic change. This framework provides a foundation for navigating that complexity and advancing meaningful, equitable innovation in healthcare.

## Note

1 Norwegian Agency for Shared Services in Education and Research (SIKT), reference number 644449.
